# Identifying Risk Factors Of A(H7N9) Outbreak by Wavelet Analysis and Generalized Estimating Equation

**DOI:** 10.3390/ijerph16081311

**Published:** 2019-04-12

**Authors:** Qinling Yan, Sanyi Tang, Zhen Jin, Yanni Xiao

**Affiliations:** 1 School of Mathematics and Information Science, Shaanxi Normal University, Xi’an 710119, China; yanqinling1222@snnu.edu.cn; 2 Complex System Research center, Shanxi University, Taiyuan 030006, China; jinzhn@263.net; 3 Department of Applied Mathematics, Xi’an Jiaotong University, Xi’an 710049, China; yxiao@xjtu.edu.cn

**Keywords:** A(H7N9), live poultry markets, wavelet analysis, Poisson regression, generalized estimating equation

## Abstract

Five epidemic waves of A(H7N9) occurred between March 2013 and May 2017 in China. However, the potential risk factors associated with disease transmission remain unclear. To address the spatial–temporal distribution of the reported A(H7N9) human cases (hereafter referred to as “cases”), statistical description and geographic information systems were employed. Based on long-term observation data, we found that males predominated the majority of A(H7N9)-infected individuals and that most males were middle-aged or elderly. Further, wavelet analysis was used to detect the variation in time-frequency between A(H7N9) cases and meteorological factors. Moreover, we formulated a Poisson regression model to explore the relationship among A(H7N9) cases and meteorological factors, the number of live poultry markets (LPMs), population density and media coverage. The main results revealed that the impact factors of A(H7N9) prevalence are manifold, and the number of LPMs has a significantly positive effect on reported A(H7N9) cases, while the effect of weekly average temperature is significantly negative. This confirms that the interaction of multiple factors could result in a serious A(H7N9) outbreak. Therefore, public health departments adopting the corresponding management measures based on both the number of LPMs and the forecast of meteorological conditions are crucial for mitigating A(H7N9) prevalence.

## 1. Introduction

An outbreak of human infections with the emerging avian influenza A(H7N9) virus occurred in China in early 2013, which caused general concern about a possible future pandemic. Humans infected with A(H7N9) virus have a high rate of morbidity and mortality (30% or so) [1,2]. By the end of 13 May 2017, the overall proportion of infected individuals (hereafter referred to as “cases”) with serious illness accounted for 58.88% (875/1486), and the proportion of fatal and dead human cases was 13.66% (203/1486). Since the first reported case in eastern China in March 2013, the outbreak of A(H7N9) has shown a characteristic spread from the south to north and even the northwest of China. Also, the A(H7N9) epidemic spread to other parts of mainland China, Hong Kong (21 cases), Macao (2 cases), Taiwan (5 cases), Malaysia (1 cases) and Canada (2 cases). Besides this, the outbreak of A(H7N9) generally shows a seasonal trend, with five major waves of outbreaks occurring to date. The rapid growth of cases in a short period of time causes panic and raises concerns about what essential factors may cause A(H7N9) outbreaks. However, due to the randomness of the outbreak area and uncertainty of multiple factors, methods of predicting A(H7N9) and then preventing and controlling the disease for public health departments remain unclear, falling within the scope of this manuscript.

Recently, several studies have been done to predict the spread of A(H7N9) viruses in humans and birds, and the potential risk factors associated with disease transmission have been estimated [1,2,3,4,5,6,7,8,9,10,11,12,13,14,15]. In particular, live poultry was identified as an important risk factor for humans being infected with A(H7N9) on the basis of the statistical analyses [2,4]. For example, most infected individuals with confirmed A(H7N9) virus infection had a history of exposure to poultry [2,4], live poultry markets (LPMs) significantly contributed to the occurrence of human infection by A(H7N9) virus [5,7,8,10,12], and LPM closures were effective in the control of human risk of avian influenza A(H7N9) virus infection [9]. Moreover, several other identified factors including sex and age [2,3,4,7,9,13], coexisting medical conditions [3], human population density, irrigated croplands, built-up land, coverage of shrub [4,5,8,12,13], location, residence type (rural or urban area), and dates of illness onset [4,9] influenced the incidence of A(H7N9). In addition, the transmission dynamical models have also been formulated to address the effects of seasonality, secular changes and environmental factors on the spread of A(H7N9), and some useful control measures have been put forward [11,14], such as timely actions to end an outbreak, careful surveillance and persistent intervention [6].

However, each of the above studies only focused on some specific factors, instead of taking all the factors into consideration comprehensively. For example, Zhang et al. focused on the impacts of temperature, humidity, sex and age on the incidence of A(H7N9), but did not take the LPMs and population density into account [13]. Fang et al. examined the relative contribution of the agro-ecological, environmental and meteorological factors on the occurrence of human A(H7N9) infection [5,10,12], but the method was not used to assess the statistical significance of individual effect variables [16]. The impacts of live poultry were considered and gender differences for infected individuals were explained, but climate factors and population density were not considered in the literature [2,3,4]. Xiao et al. only focused on addressing the effectiveness of control measures without considering the temperature and precipitation (humidity) [6,11,14]. Bui et al. considered the animal environment case data and spatial precision, but they only focused on domestic factors, including neither LPMs nor the specific transmission dynamics [15]. Fortunately, the relationship between A(H7N9) prevalence and the main environmental factors was considered comprehensively [17]. Nevertheless, due to too many environmental variables being included in the model, environmental factors of A(H7N9) human infection have not been clearly identified.

Therefore, in order to take all factors into consideration and identify the key factors that influence A(H7N9) prevalence, and to explain these controversial conclusions from different studies, we will focus on the following issues: (i) weekly reported data sets from March 2013 to May 2017 in China (named long-term data—if the length of the time series is less than one year, then it is here called short-term data) are used to address multiple factors including temperature, total precipitation (humidity), and LPMs on the incidence of A(H7N9). As a result, the types of data are complex, including both longitudinal and cross-sectional data, which undoubtedly brings challenges for data analysis, model fitting and prediction; (ii) generalized estimating equations (GEE) are employed to reveal the key uncertainties and to identify the most important factors; (iii) the effects of the interaction of temperature and precipitation (humidity) and media coverage on the incidence of A(H7N9) are discussed; (iv) methods of preventing and controlling the disease for public health departments for emerging infectious A(H7N9) outbreaks, which is a key public health issue.

The rest of this paper is organized as follows. First of all, we introduce all data sets used in this study, including weekly reported human cases of A(H7N9) from the department of the Centre for Health Protection and the Global Animal Disease Information System, meteorological factors, the number of LPMs, population density and media coverage. We then use a statistical description for the collected data at the individual level and map the annual cumulative number of A(H7N9) cases and the number of LPMs in affected regions by GIS to characterize the spatial–temporal distribution of A(H7N9) outbreaks. Further, wavelet analysis is used to decompose the time series (A(H7N9) and meteorological factors) into the time–frequency space and then detect the variation of periodicity between them over time. Moreover, a Poisson regression model which explores the relationship among A(H7N9) cases and measurements of meteorological factors, the number of LPMs, population density and media coverage is introduced and investigated, and the GEE method is employed to obtain the best-fit parameter values and consequently identify the risk factors of A(H7N9) outbreak.

## 2. Materials and Methods

### 2.1. Data Source

#### 2.1.1. Surveillance for A(H7N9)

**Figure 1:**
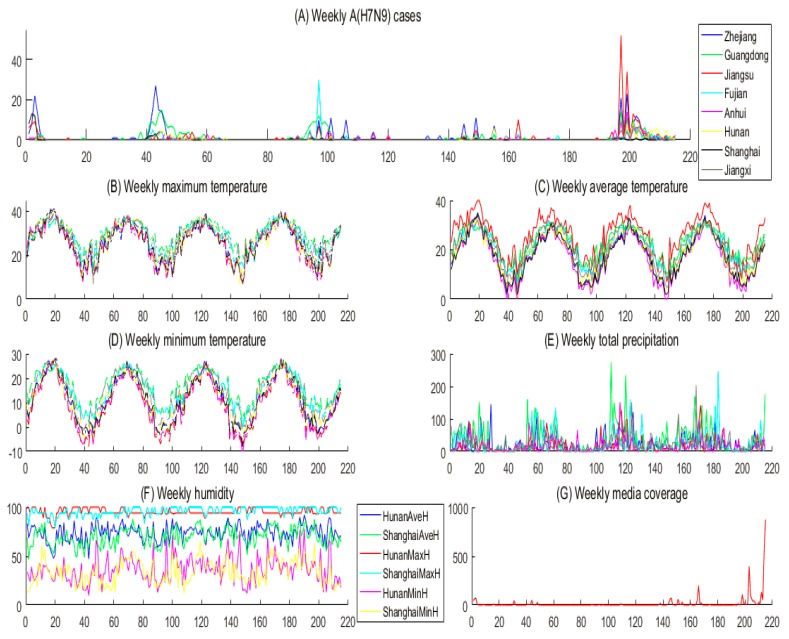
Time series plots of weekly reported human cases of A(H7N9), meteorological factors and media coverage from 31 March 2013 to 13 May 2017 for the provinces Zhejiang, Guangdong, Jiangsu, Fujian, Anhui, Hunan, Shanghai and Jiangxi. (**A**) Weekly reported human cases of A(H7N9); (**B**) weekly maximum temperature; (**C**) weekly average temperature; (**D**) weekly minimum temperature; (**E**) weekly total precipitation for Zhejiang, Guangdong, Jiangsu, Fujian, Anhui and Jiangxi; (**F**) maximum relative humidity, average relative humidity, minimum relative humidity for Hunan and Shanghai; and (**G**) the number of weekly news items relevant to A(H7N9).

Data regarding weekly reported human cases of A(H7N9) in this study were obtained from the department of the Centre for Health Protection [18] and the Global Animal Disease Information System [19] in mainland China (including 23 provinces, 4 municipalities, and 5 autonomous regions), Hong Kong, Macao and Taiwan, from 31 March 2013 to 13 May 2017, as shown in Figure 1A. Individual case information includes the geographic location, age, gender, clinical condition, date of report and whether or not the person ever had contact with live poultry. A confirmed case was defined according to the World Health Organization criteria [20] and national authorities.

#### 2.1.2. Data for Other Variables

**Figure 2:**
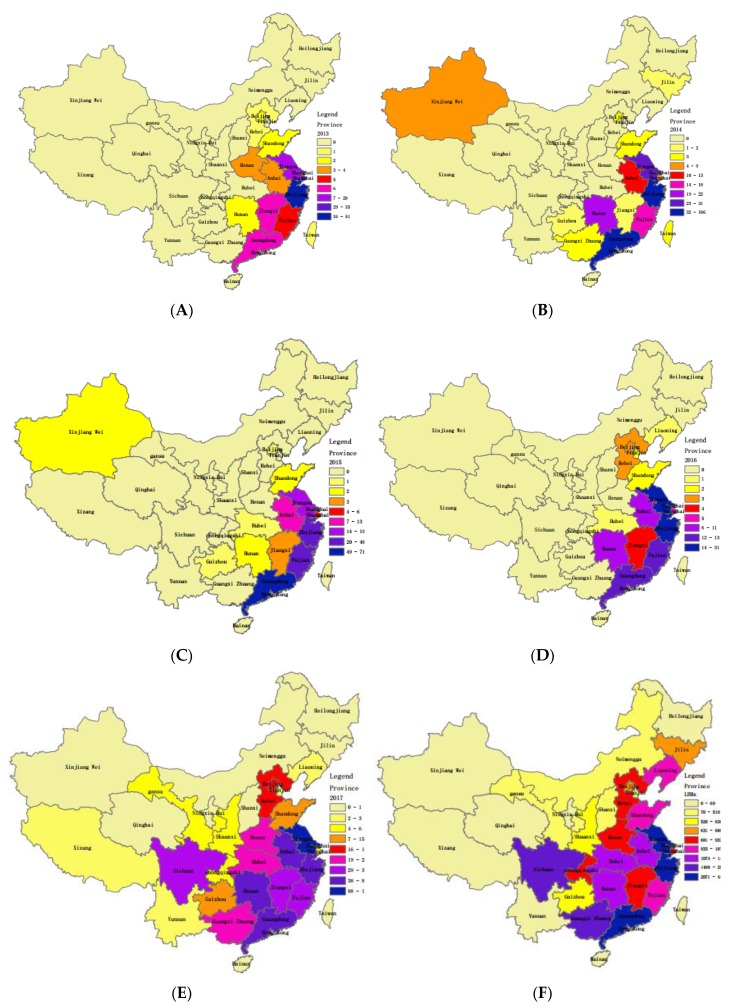
The map of reported human cases of A(H7N9) prevalence and the number of live poultry markets (LPMs) in China. (**A**)–(**E**) Distribution of cumulative numbers of A(H7N9) cases each year from 31 March 2013 to 13 May 2017 in mainland China (including 23 provinces, 4 municipalities, and 5 autonomous regions), Hong Kong, Macao and Taiwan. The number of cases at the region level is reflected by colored gradients, as shown in the legend. (**F**) Distribution of LPMs for regions in 2017. The map was plotted in ArcGIS 10.2 software (Esri, Redlands, CA, USA).

Data concerning meteorological factors, the number of LPMs and population of each region, and media coverage were collected from websites. The data of meteorological variables including weekly temperature (maximum temperature, average temperature and minimum temperature) and weekly total precipitation (or maximum relative humidity, average relative humidity and minimum relative humidity) were obtained from Weather Underground [21] during the study period, as shown in Figure 1B–F. We extracted the data associated with LPMs with information on the name and location in each city from Baidu Map API (Baidu, Beijing, China) using the JavaScript language. The search terms in JavaScript language included “city name”, “live poultry/poultry” and “trade/wholesale/retail”. Then, in order to ensure the accuracy of the obtained data, we sorted and screened results manually. The population size of each province/autonomous region was obtained from the National Bureau of Statistics of China [22], from which population densities were calculated based on the area of each region, as shown in Figure 2. In addition, the number of weekly news items relevant to the A(H7N9) was calculated from Baidu News [23], as shown in Figure 1G.

### 2.2. Thematic Map of A(H7N9)

To characterize the spatial–temporal distribution of A(H7N9) outbreaks, a thematic map of the cumulative A(H7N9) cases each year in affected regions was produced in ArcGIS 10.2 software (Esri, Redlands, CA, USA) during the study period; also, the LPMs were mapped. Due to the prevalence in provinces including Zhejiang, Guangdong, Jiangsu, Fujian, Anhui, Hunan, Shanghai, Jiangxi being relatively severe, in the following, we mainly focus on investigating the impact of meteorological and other factors on the A(H7N9) prevalence in these provinces.

### 2.3. Wavelet Analysis

Wavelet analysis can be used to decompose a time series into the time–frequency space and then detect the variation of periodicity over time [24]. Therefore, wavelet coherence, one class of the wavelet transform method, was used to examine the association of two time series in time and frequency; i.e., whether two time series oscillate simultaneously. The level of wavelet coherence indicates the capability of one time series to predict the other, and the phase relationship between the series indicates the expected causality links [25]. The detailed descriptions of the method are shown in Supplementary Materials A.

Since the A(H7N9) virus is a low pathogenic avian influenza A virus and does not cause identifiable illness or death in poultry, and the source of A(H7N9) virus infection in the confirmed cases who had exposure to animals cannot be verified, only laboratory testing can identify poultry infections; as an additional challenge, only a small number of identifiable infected individuals have had contact with live birds [2]. However, many studies have identified visiting LPMs as a risk factor, and Yu et al. [9] estimated that the closure of LPMs reduced the mean daily number of A(H7N9) virus infections in the four most affected cities (including Shanghai, Hangzhou, Huzhou and Nanjing) by 97% to 99% [9]. This further confirms that it is necessary to consider LPMs as a risk variable to study their impact on A(H7N9) outbreaks.

### 2.4. Poisson Regression

Poisson regression models are widely used to examine the relationship between time-series count data and ambient environmental factors, including weather and other time-varying confounders [26,27]. Therefore, the relationships among reported human cases of A(H7N9) and measurements of meteorological factors, the number of LPMs, population density and media coverage were explored by Poisson regression. We assumed the reported human cases of A(H7N9) follow a Poisson distribution with mean $E\left(Y_{i,t}\right)$ (the $Y_{i,t}$ is the A(H7N9) observations in the *i*-th province on the *t*-th week per annum from the 31 March 2013). Furthermore, in order to account for the time-delay effect of temperature, precipitation and humidity, we used cross-correlation analysis to determine the appropriate time lag to be used in our model [28]. The results show that there is a statistically significant cross-correlation (r = −0.232) between the temperature and reported human cases of A(H7N9) at a lag of 3 weeks, but no statistically significant cross-correlation between the precipitation/humidity and reported human cases of A(H7N9). Additionally, we used a trigonometric model-harmonic cycle plus a linear function ($c_{0}+c_{1}t\sin\frac{2\pi }{52}t+c_{2}t\cos\frac{2\pi }{52}t$) to capture the trend and seasonal pattern in the observed weekly data [29]. Compared with the magnitude of temperature, precipitation, and media coverage, the magnitudes of the population density (pop) and the number of live poultry markets (LPMs) are very large. In order to increase the estimation accuracy of the parameters, we used a logarithmic change in population density and LPMs. Thus, we obtain the following model:

(1) $$\mathrm{Log}\left[E\left(Y_{i,t}\right)\right]=\alpha +c_{1}t\sin\frac{2\pi }{52}t+c_{2}t\cos\frac{2\pi }{52}t+\beta_{1}T_{i,t-3}+\beta_{2}P_{i,t}+\beta_{3}T_{i,t-3}P_{i,t}+\delta log_{10}\left(pop_{i}\right)\;+\gamma log_{10}\left(LPMs_{i}\right)+\lambda M_{t},i=1,\cdots ,8;t=1,\cdots ,52.$$

where α is a constant parameter (including $c_{0}$); $T_{i,t-3},P_{i,t}$ and $T_{i,t-3}P_{i,t}$ refer to temperature, precipitation and their interaction effects in the *i*-th province on the (*t*−3)-th (*t*-th) week, respectively; $pop_{i}$ and $LPMs_{i}$ refer to population density and the total number of LPMs in the *i*-th province, respectively; and $M_{t}$ refers to the number of media reports on the *t*-th week.

### 2.5. Generalized Estimating Equation

The generalized estimating equation (GEE) has been widely used to estimate parameters of the generalized linear model based on longitudinal data sets [30,31]. The GEE allows us to specify a working correlation matrix that accounts for the form of the within-subject correlation of responses on dependent variables of the exponential distribution family [30,31,32]. Besides this, one advantage of GEE is that it is not necessary to specify the working correlation matrix correctly to obtain a consistent and asymptotically Gaussian estimation for parameters. Therefore, the GEE was employed to estimate parameters of model (1). Furthermore, considering that there are five sorts of working correlation matrix (independent, exchangeable, first-order autoregression (AR(1)), M-dependent and unstructured), the quasi-likelihood under independence model criterion (QIC) was employed to select the appropriate correlation. The working correlation matrix with the minimum QIC value will be optimal. 

## 3. Results

### 3.1. Distribution Characteristics of Reported A(H7N9) Cases and LPMs

A total of 1486 cases infected with A(H7N9) virus were confirmed by laboratory tests and reported from the department of the Centre for Health Protection since its announced emergence on 31 March 2013. The statistical description for the collected data at the individual level is shown in Table 1, from which we can see that, for the long-term data, the male patients also predominated in number over female infected individuals, with a sex ratio of approximately 2:1; the majority of cases are in middle-aged and elderly individuals, making up about 46.30% and 24.70% of the total number, respectively. Besides this, the middle-aged and elderly infected individuals accounted for a large part of the detected male population, with the proportion being as high as 81.79%.

In addition, we draw time series of weekly reported human cases of A(H7N9), meteorological factors and media coverage from 31 March 2013 to 13 May 2017 in Figure 1, from which we can see that almost all meteorological factors have a one-year cycle, which is similar to the variations shown in the A(H7N9) cases. Moreover, there are five A(H7N9) outbreak peaks during the period from 31 March 2013 to 13 May 2017, roughly within the time intervals [0, 4], [41, 49], [93, 103], [145, 149] and [195, 206], respectively. Moreover, Figure 1 shows that the weekly maximum (average, minimum) temperature, total precipitation, and average (minimum) relative humidity reach their minimums within those intervals, which indicates that the low temperature and dry climate are associated with the incidence of A(H7N9). However, media coverage may have little or no correlation with the incidence of A(H7N9).

By using GIS technologies, maps of A(H7N9) cases for each year and LPMs were created for mainland China, as shown in Figure 2. As can be seen in the maps in Figure 2A–E, the A(H7N9) prevalence of infection became worse, except for in 2016, and the spreading trend was from southeast to northwest. In particular, the epidemic was especially severe in 2017, spreading from southeast to north to Liaoning, west to Tibet, and northwest to Gansu. Provinces with relatively severe epidemics were Zhejiang, Guangdong, Jiangsu, Fujian, Anhui, Hunan, Shanghai and Jiangxi. By 13 May 2017, the cumulative number of A(H7N9) cases for the province with the most severe A(H7N9) prevalence (Zhejiang) had reached 307, and the province with the lowest number of reported cases within the above 8 provinces was Jiangxi (52 cases). Note that it follows from Figure 2E,F that the spatial distributions of the A(H7N9) prevalence and LPMs have similar patterns, which indicates that the number of LPMs and A(H7N9) cases may have relevance. To confirm this, the correlation between the cumulative number of A(H7N9) cases and the number of LPMs of each province in 2017 was analyzed, and the Pearson correlation coefficient was 0.826, which was statistically significant. However, the Pearson correction coefficients were not calculated due to the small numbers of reported cases in each province from 2013 to 2016.

### 3.2. Wavelet Coherence between Weekly Reported Human Cases of A(H7N9) and Meteorological Factors

**Figure 3:**
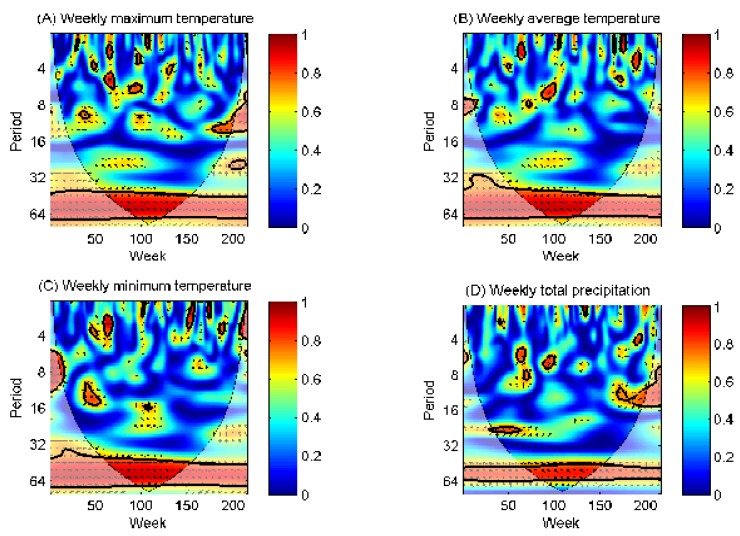
The wavelet coherence between weekly reported human cases of A(H7N9). (**A**) Weekly maximum temperature; (**B**) weekly average temperature; (**C**) weekly minimum temperature; and (**D**) weekly total precipitation for Zhejiang province.

To explore whether the meteorological factors affect the strong seasonality of A(H7N9) cases for the above eight provinces, all data sets were normalized before employing wavelet analyses. Wavelet coherence was used to examine the connection between A(H7N9) cases and meteorological factors, especially to depict the phase relationships between these series, which can reveal the expected causality links. The results for the Zhejiang province are shown in Figure 3.

We found that weekly maximum temperature, average temperature, minimum temperature and total precipitation presented a consistently significant coherence with weekly A(H7N9) cases between the 32- and 64-week (approximately annual cycle) band (*p* < 0.05) throughout the study period. At the annual time scale, these factors were negatively correlated with A(H7N9) cases, as shown in Figure 3. For lunar time scales, such as periods shorter than 32 weeks, significant coherence between A(H7N9) cases and meteorological factors were incompletely consistent but mostly negative.

The strong correlation between meteorological factors and A(H7N9) cases on an annual scale can be depicted based on the fact that the total number of A(H7N9) weekly reported cases in hot and rainy summers is significantly less than that in cold and dry winters for the Zhejiang province [33], which was partially addressed in previous studies [34]. The negative correlation indicates that rising temperatures can help suppress the spread of the avian influenza virus, which is more likely to survive in a low-temperature and humid environment. For example, previous studies and experiences in fighting influenza have shown that avian influenza virus can survive for a week at low temperature in the stool, and can survive for a month in 4 °C water; as the temperature increases, the virus will gradually lose its activity.

Similarly, we analyzed the connection between A(H7N9) cases and meteorological factors for another seven provinces, as shown in Supplementary Figures S1–S7. The main results indicate that the impact of temperature on A(H7N9) prevalence in these provinces (including Guangdong, Jiangsu, Fujian, Anhui and Shanghai) is similar to that in Zhejiang; i.e., both on the annual scale and lunar scales, the temperature can have significant effects on the A(H7N9) weekly reported cases. However, the effects of the temperature on the A(H7N9) prevalence for Jiangxi and Hunan can only be found at some short time scales. Moreover, the effects of total precipitation (relative humidity) on the A(H7N9) prevalence are quite different.

The reasons for the above difference could be useful in mitigating A(H7N9) outbreaks, and are as follows: (i) the quite different climate factors in different geographical regions; (ii) the different economic and traffic levels—for instance, Guangdong, Shanghai etc. are coastal provinces/cities, while Jiangxi and Hunan are landlocked provinces, which leads to a big difference in breeding, transporting, slaughtering and processing modes of live poultry; (iii) the number of LPMs is very large in Zhejiang and Guangdong, while it is small in Jiangxi and Hunan; (iv) the numbers of weekly reported cases in Jiangxi and Hunan provinces were very small within a short period, which may result in some deviations for long-term data analyses.

### 3.3. Main Results Based on GEE

The GEE was used to analyze the relationship between reported human cases of A(H7N9) and measurements of meteorological factors, the number of LPMs, population density and media coverage by Poisson regression, and a working correlation matrix with AR(1) was selected for the minimum QIC value. The results are shown in Table 2. They present the point estimators of GEE ($\hat{\beta }$), the corresponding exponential quadratic ($e^{\hat{\beta }}$) and the confidence interval $\left(e^{{\hat{\beta }}_{LB}},\;e^{{\hat{\beta }}_{UB}}\right)$ of $\hat{\beta }$ for model (1).

From Table 2, we conclude that the Poisson regression model (1) based on the A(H7N9) cases identifies the number of LPMs as the most significant risk factor according to Wald’s test [35]. The rate is 16.510 (95% CI [5.404, 50.442]), meaning that the expected number of A(H7N9) infections in humans increases 16.51 for each additional logarithm of the number of LPMs. In other words, if the number of LPMs decreases by 10%, 20%, 50%, 70% and 90%, then the expected number of A(H7N9) infection in humans will decrease by 0.755 (95% confidence interval (CI) [0.247, 2.308]), 1.600 (95% CI [0.524, 4.888]), 4.970 (95% CI [1.627, 15.185]), 8.633 (95% CI [2.828, 26.375]) and 16.51 (95% CI [5.404, 50.442]), respectively. Besides this, the weekly average temperature has a statistically negative effect on incidence of A(H7N9), with a rate of 0.339 (95% CI [0.272, 0.422]); that is, the expected number of A(H7N9) infections in humans decreases by 0.722 (95% CI [0.579, 0.899]) as the temperature increases by 1 °C at an average temperature level of 18.6 °C. Moreover, the trend and seasonality of the weekly time series are two significant risk factors. Furthermore, the results reveal that the interaction between average temperature and total precipitation, the total precipitation, population density and media coverage have a certain impact on A(H7N9) prevalence but are not statistically significant. 

Based on the above sensitivity analyses, we can conclude that both the number of LPMs and temperature have a great impact on the A(H7N9) prevalence. Besides this, seasonal changes also have a certain impact on the A(H7N9) prevalence. Therefore, in order to control the A(H7N9) prevalence and to take into account the comprehensive benefits of the economy, government departments can take measures to close LPMs and monitor and provide an early warning of environmental factors (including temperature) which can effectively change people’s behavior in areas with a high risk of an epidemic.

## 4. Discussion

It is well established in the epidemiological literature that some risk factors have effects on the outcome of A(H7N9) infection (based on short-term data). However, the combination of the GIS, wavelet analysis and GEE (based on long-term data) is important to explore and explain the following possible causes for the distribution characteristics of A(H7N9) cases: (i) personal immune levels for the A(H7N9) virus may be affected by the nature of the work undertaken [2,4]; (ii) the negative impact of temperature on the A(H7N9) prevalence was confirmed based on long-term observation data; and (iii) the number of LPMs is the most significant factor for outbreaks of A(H7N9). These results show the key risk factors, and thus aid in designing public health communication strategies and disease mitigation measures for health-policy-makers.

Initially, we infer that personal immune level for the A(H7N9) virus may be affected by the nature of the person’s work based on the characteristics they have in common with others at the individual level and the spatial-temporal distribution of A(H7N9) cases. For example, it is obvious that the number of males under 18 years old was equal to or even smaller than that of women, while the number of males over 18 years old was about 2 times as large as that of females in Table 1. Besides this, an interesting phenomenon is observed in which males accounted for the majority of A(H7N9) infected individuals, and most of the males are middle-aged and elderly. The main reason for the above phenomenon is that, while it may be not inherently true that males are “less immune than females”, males are more likely to be infected with A(H7N9) virus because of their careers, work environment, physical fitness, smoking behavior, etc. For example, males may be more likely to engage in poultry farming, trafficking, selling, slaughtering, processing and so on; males who are smokers are susceptible individuals because of their pulmonary dysfunction associated with smoking; middle-aged and elderly infected individuals may frequently go shopping in LPMs; the majority of people who run LPMs may be middle-aged and elderly people; and elderly persons may have an increased risk of coexisting illnesses and are thus more susceptible to severe disease than younger persons.

Next, the negative impact of temperature on the A(H7N9) prevalence was confirmed based on long-term observation data by wavelet analysis and GEE method. Wavelet coherence between temperature and A(H7N9) cases in the time–frequency space show that the temperature was negatively correlated with A(H7N9) cases both at an annual scale and lunar scale (mostly negative), with a correlation size varying in different provinces except for in Jiangxi and Hunan. The GEE analysis results show that the temperature has a significantly negative effect on the A(H7N9) prevalence based on the overall long-term data. Furthermore, sensitivity analysis reveals that the expected number of A(H7N9) infection in humans decreases by 0.722 (95% CI [0.579, 0.899]) as the temperature increases by 1 °C at an average temperature level of 18.6 °C.

Therefore, we conclude that the main results obtained here are different from the previous studies into the effects of LPMs and temperature [8,12,13,33] since long-term data are employed; i.e., the impact factors for the A (H7N9) prevalence are manifold instead of single, and the number of LPMs and weekly average temperature are the two factors that most significantly positively and negatively affect the A(H7N9) prevalence, respectively. This reveals the importance of data integrity, using wavelet techniques for long-term data and considering the impact of the seasonality in this study. Furthermore, these results show that the A(H7N9) virus has a great risk of transmission at the appropriate temperature range, which indicates that the monitoring and early warning of environmental factors (including temperature) can effectively change people’s behavior in areas with a high risk of epidemics and consequently help us to mitigate A(H7N9) human infection.

More importantly, data analyses depict that the most serious outbreaks for A(H7N9) occurred at the time about 20 days before and after the Spring Festival each year for the eight provinces with the highest number of LPMs (especially in Zhejiang, Guangdong, Jiangsu province, where China’s economic development zone and the largest poultry processing plants are), which has been confirmed by the report of the Food and Agriculture Organization of the United Nations [36]. Therefore, to mitigate emerging infectious diseases including A(H7N9), the following control strategies could be proposed: (i) because the number of LPMs is the most significant factor for outbreaks of A(H7N9), the management of LPMs during holiday seasons should be strengthened, such as by strictly checking poultry and prohibiting infected poultry; (ii) based on the result that personal immune level for the A(H7N9) virus may be affected by the nature of a person’s work, live poultry-breeding personnel, transporting personnel, and slaughtering personnel and workers should be subject to good protective measures, including wearing protective equipment, cleaning the environment in a timely manner, and good personal hygiene; (iii) since the most serious outbreaks for A(H7N9) occurred at the time about 20 days before and after the Spring Festival each year, and as LPMs are the most significant factor for outbreaks, some behavior-changing measures should be taken for the public, such as people trying to avoid LPMs and paying attention to their diet (eating high-temperature processed meat and poultry related products) during the Spring Festival. On the other hand, Chinese eating habits can be easily changed due to media reporting on the severity of infectious disease, and consequently people could change their behavior and not visit or reduce the frequency of their visits to poultry markets during the high-risk period of disease transmission. Therefore, under properly guided media publicity by the government, the risk of transmission can be effectively mitigated.

However, when we omit the temperature and total precipitation (in Figure 1B–E) in the time period of the five peaks corresponding to the maximum number of A(H7N9) cases in Figure 1A (as shown in Supplementary Figure S8), we find that both the temperature and the number of A(H7N9) cases were the lowest in 2016; the possible reasons for this are as follows: (i) strong measures to restrict and prohibit live poultry trading were implemented. Taking Guangzhou as an example, live poultry trading was held for three days before and after the Spring Festival [37,38,39]; (ii) the Agriculture, Trade, Health, Food and Drug Administration and other departments have jointly supervised LPMs [38]; (iii) some departments have sought new development opportunities such as developing chilled chicken. For example, Shenzhen has carried out the operation of “centralized slaughtering, cold chain distributing, fresh listing” and so on [40]. Unfortunately, on the one hand, live poultry trading was frequent, and there were loopholes in the supervision [38]. On the other hand, chilled chicken was developed and has begun to take shape in the first and second-tier cities only, but was still not present in the third or fourth-tier cities [40]. These could be the two main reasons for the A(H7N9) outbreak in 2017 being persistent in underdeveloped areas.

Our study has several limitations. First, as the collected data lack detailed information from all infected individuals regarding exposures (including the times, frequency, intensity, and duration of exposures) and coexisting illnesses (such as coronary heart disease, cerebrovascular disease, etc.), the impact of these factors on the incidence of A(H7N9) at the individual level were not evaluated. Second, since we do not know when and how the LPMs might be closed, the effects of closing LPMs on A(H7N9) prevalence in the long run cannot be exactly analyzed.

## 5. Conclusions

This study presented a novel methodology by studying A(H7N9) prevalence on the individual level and population level by GIS, wavelet analysis, and GEE, and the main results reveal that a combination of individual and population levels is beneficial for analyzing the impact of possible risk factors on disease outbreaks. It demonstrated that A(H7N9) prevalence was affected by a number of factors; in particular, the number of LPMs has the most significant impact on A(H7N9) prevalence, followed by the effect of temperature. Furthermore, the interaction effects between average temperature and total precipitation, the total precipitation, population density and media coverage have impacts on A(H7N9) prevalence, but their effects are not statistically significant. Therefore, for mitigating and controlling A(H7N9) prevalence, public health departments should take corresponding management measures based on the number of LPMs and the forecast of the temperature by meteorological observatories.

## Figures and Tables

**Table 1:** The statistical description for the collected data at the individual level.

Variable	No. (2013)	No. (2014)	No. (2015)	No. (2016)	No. (2017)	Total no.
Sex						
Male		218	148	84	456	906
Female		105	63	39	196	403
Unknown	147	0	3	12	15	177
Age (Total (Male+ Female))						
0~6		14(4 + 10)	14(6 + 8)	0(0 + 0)	4(1 + 3)	32(11 + 21)
7~17		7(3 + 4)	2(1 + 1)	1(0 + 1)	4(3 + 1)	14(7 + 7)
18~40		62(46 + 16)	25(14 + 11)	19(15 + 4)	104(72 + 32)	210(147 + 63)
41~65		142(99 + 43)	117(84 + 33)	63(39 + 24)	366(256 + 110)	688(478 + 210)
≥66		98(67 + 21)	53(43 + 10)	40(29 + 11)	176(124 + 52)	367(263 + 104)
Unknown	147	0	3	12	13	175

**Table 2:** Parametric estimated value based on the collected data including A(H7N9) cases, meteorological factors, the number of LPMs, population density and media coverage and Poisson regression model.

Parameter	$\hat{\beta }$	$e^{\hat{\beta }}$	$e^{{\hat{\beta }}_{LB}}$	$e^{{\hat{\beta }}_{UB}}$
α	−9.149 *	0.000	1.298 $\times 10^{-6}$	0.009
$c_{1}$	0.022 *	1.022	1.008	1.036
$c_{2}$	−0.008 *	0.992	0.985	1.000
$\beta_{1}$	−1.082 *	0.339	0.272	0.422
$\beta_{2}$	−0.157	0.845	0.730	1.000
$\beta_{3}$	−0.164	0.849	0.683	1.055
δ	−0.405	0.667	0.256	1.737
γ	2.804 *	16.510	5.404	50.442
λ	0.001	1.001	1.001	1.001

* Represents that the parameter has a statistically significant different from zero.

## Supplementary Materials

The following are available online at https://www.mdpi.com/1660-4601/16/8/1311/s1, A. Wavelet analyses (A.1. Wavelet Power Spectrum; A.2. Wavelet coherence and phase; A.3. Wavelet Power Spectrum; A.4. Graphical description). B: Figures S1–S8.

## References

[1] Liu H.H., Li T., Zheng Y.F. (2013). Poor responses to oseltamivir treatment in a patient with influenza A (H7N9) virus infection. Emerg. Microbes Infect..

[2] Li Q., Zhou L., Zhou M.H. (2014). Epidemiology of Human Infections with Avian Influenza A(H7N9) Virus in China. N. Engl. J. Med..

[3] Gao H.N., Lu H.Z., Cao B. (2013). Clinical Findings in 111 Cases of Influenza A(H7N9) Virus Infection. N. Engl. J. Med..

[4] Cowling B.J., Jin L.M., Lau E.H.Y. (2013). Comparative epidemiology of human infections with avian influenza A(H7N9) and A(H5N1) viruses in China. Lancet.

[5] Fang L.Q., Li X.L., Liu K. (2013). Mapping Spread and Risk of Avian Influenza A (H7N9) in China. Sci. Rep..

[6] Xiao Y.N., Sun X.D., Tang S.Y. (2014). Transmission potential of the novel avian influenza A(H7N9) infection in mainland China. J. Theor. Biol..

[7] Cowling B.J., Freeman G., Wong J.Y. (2013). Preliminary inferences on the age-specific seriousness of human disease caused by avian influenza A(H7N9) infections in China, March-April 2013. Eur. Surveil.

[8] Fuller T., Havers F., Xu C.L. (2014). Identifying areas with a high risk of human infection with the avian influenza A (H7N9) virus in East Asia. J. Infect..

[9] Yu H.J., Wu J.T., Cowling B.J. (2014). Effect of closure of live poultry markets on poultry-to-person transmission of avian influenza A H7N9 virus: An ecological study. Lancet.

[10] Gilbert M., Golding N., Zhou H. (2014). Predicting the risk of avian influenza A H7N9 infection in live-poultry markets across Asia. Nat. Commun..

[11] Hsieh Y.H., Wu J.H., Fang J. (2014). Quantification of Bird-to-Bird and Bird-to Human Infections during 2013 Novel H7N9 Avian Influenza Outbreak in China. PLoS ONE.

[12] Li X.L., Yang Y., Sun Y. (2015). Risk Distribution of Human Infections with Avian Influenza H7N9 and H5N1 virus in China. Sci. Rep..

[13] Zhang Y., Feng C., Ma C.N. (2015). The impact of temperature and relative humidity measures on influenza A(H7N9) outbreaks-evidence from China. Int. J. Infect. Dis..

[14] Lin Q.Y., Lin Z.G., Chiu A.P.Y. (2016). Seasonality of Influenza A(H7N9) Virus in China-Fitting Simple Epidemic Models to Human Cases. PLoS ONE.

[15] Bui C.M., Gardner L., MacIntyre R., Sarkar S. (2017). Influenza A H5N1 and H7N9 in China: A spatial risk analysis. PLoS ONE.

[16] Martin V., Pfeiffer D.U., Zhou X.Y. (2011). Spatial distribution and risk factors of highly pathogenic avian influenza (HPAI) H5N1 in China. PLoS Pathog..

[17] Xu M., Cao C.X., Li Q., Jia P., Zhao J. (2016). Ecological Niche Modeling of Risk Factors for H7N9 Human Infection in China. Int. J. Environ. Res. Public Health.

[18] Centre for Health Protection. http://www.chp.gov.hk.

[19] EMPRES Global Animal Disease Information System. http://empres-i.fao.org/eipws3g.

[20] World Health Organization. http://www.who.int/csr/resources/publications/surveillance/.

[21] The Weather Underground. https://www.wunderground.com.

[22] Population Census Office under the State Council & Department of Population and Employment Statistics, National Bureau of Statistics of China, Tabulation on the 2016 1% Population Survey of the People’s Republic China by Regions. http://www.stats.gov.cn/tjsj/ndsj/2016/indexch.htm.

[23] Baidu News. http://news.baidu.com.

[24] Torrence C., Compo G.P. (1998). A practical guide to wavelet analysis. Bull. Am. Meteorol. Soc..

[25] Grinsted A., Moore J.C., Jevrejeva S. (2014). Application of the cross wavelet transform and wavelet coherence to geophysical time series. Nonlinear Process. Geophys..

[26] McCullagh P., Nelder J.A. (1989). Generalized Linear Models.

[27] Dominici F., Sheppard L., Clyde M. (2003). Health effects of air pollution: A statistical review. Int. Stat. Rev..

[28] William W.S. (2006). Time Series Analysis: Univariate and Multivariate Methods. Transfer Function Models.

[29] Montgomery D.C., Jennings C.L., Kulahci M. (2008). Introduction to Time Series Analysis and Forecasting.

[30] Zeger S.L., Liang K.Y. (1986). Longitudinal data analysis for discrete and continuous outcomes. Biometrics.

[31] Liang K.Y., Zeger S.L. (1986). Longitudinal data analysis using generalized linear models. Biometrika.

[32] Zeger S.L., Liang K.Y. (1992). An overview of methods for the analysis of longitudinal data. Stat. Med..

[33] Baike. https://baike.so.com/doc/4535588-4745807.html.

[34] Lowen A.C., Mubareka S., Steel J., Palese P. (2007). Influenza virus transmission is dependent on relative humidity and temperature. PLoS Pathogens.

[35] Hauck W.W., Donner A. (1977). Wald’s test as applied to hypotheses in logit analysis. J. Am. Stat. Assoc..

[36] Food and Agriculture Organization of the United Nation Chinese-Origin H7N9 Avian Influenza Spread in Poultry and Human Exposure-Qualitative Risk Assessment Update. www.fao.org/3/i8705en/I8705EN.

[37] Zhejiang news. http://zjnews.zjol.com.cn/.

[38] NetEase News. http://news.163.com.

[39] Eastday.com. http://sh.eastday.com/m/.

[40] Chyxx. http://www.chyxx.com.

