# Supplementary material for: Identifying Risk Factors Of A(H7N9) Outbreak by Wavelet Analysis and Generalized Estimating Equation
Source: Int J Environ Res Public Health. 2019 Apr 12;16(8):1311. doi: 10.3390/ijerph16081311 (PMC6518036; doi:10.3390/ijerph16081311)
Supplement: Supplementary file 1 [file ijerph-16-01311-s001.pdf]

International Journal of
Environmental Research and Public Health

MDPI

Supplementary Materials

Identifying Risk Factors Of A(H7N9) Outbreak by Wavelet Analysis and Generalized Estimating Equation

Qinling Yan [1], Sanyi Tang [1,*], Zhen Jin [2] and Yanni Xiao [3]

1 School of Mathematics and Information Science, Shaanxi Normal University, Xi'an, P.R.China; yanqinling1222@snnu.edu.cn
2 Complex System Research center, Shanxi University, Taiyuan, P.R.China; jinzhn@263.net
3 Department of Applied Mathematics, Xi'an Jiaotong University, Xi'an, P.R.China; yxiao@xjtu.edu.cn
* Correspondence: sytang@snnu.edu.cn or sanyitang219@hotmail.com

A. Wavelet analyses

Wavelet analysis is becoming a common tool for analyzing localized intermittent oscillations in a time series. By decomposing a time series into time-frequency space, the cross wavelet transform and wavelet coherence are able to analyze whether regions in time frequency space with large common power exist a consistent phase relationship, and consequently are suggestive of causality between the time series.

A.1. Wavelet Power Spectrum

The wavelet power spectrum is assumed as a function of wavelet transform, which is the convolution of a discrete sequence (x_n) and Morlet wavelet (ψ). We can obtain the following transform at time $i\delta t$ on a scale s of a discrete time series x_j of length N with a sampling interval by translating the wavelet in time and computing the convolution [1-3]:

$$WT_i(s)=\sum_{j=1}^{j=N} x_j\psi^*[(j-i)\,\delta t/s] \quad \text{(A1)}$$

where the (*) indicates the complex conjugate. In addition, the $WT_i(s)$ can divided into the real part ($R\{WT_i(s)\}$) and imaginary part ($I\{WT_i(s)\}$), or phase ($\tan^{-1}[I\{WT_i(s)\}/R\{WT_i(s)\}]$) and amplitude ($|WT_i(s)|^2$). We can construct a picture showing both the amplitude of any features versus the scale by varying the wavelet scale s and translating along the localized time index i, and further showing how this amplitude varies with time [1-4]. Wavelet power spectrum can be defined by the squared wavelet transform:

$$WPT_i(s)=|WT_i(s)|^2 \quad \text{(A2)}$$

A.2. Wavelet coherence and phase

For the two wavelet transforms $WT_i^X(s)$ and $WT_i^Y(s)$ of time series X and Y, the cross-wavelet spectrum is defined as $WCO_i(s)=WT_i^X(s)WT_i^{Y*}(s)$ with the cross-wavelet power $|WCS_i(s)|$ (where $WT_i^{Y*}(s)$ is the complex conjugate of $WT_i^Y(s)$. Then the wavelet coherence is defined as the square of the cross-spectrum normalized by the individual power spectra:

$$WCO_i(s)=\frac{|WCS_i(s)|}{(WPT_i^X(s)WPT_i^{Y*}(s))^{1/2}} \quad \text{(A3)}$$

Thus, the $WCO_i(s)$ as a function of frequency measures the cross-correlation between two time series and varies between 0 and 1. In particular, the value of 1 means a linear relationship between X and Y around time $i\delta t$ on a scale s and 0 indicates uncorrelated [1,3]. Similar to the definition of wavelet transform phase, the coherence phase is defined as $\tan^{-1}[I\{WCS_i(s)\}/R\{WCS_i(s)\}]$.

A.3. Wavelet Power Spectrum

The confidence interval is defined as the probability that the true wavelet power at a certain time and scale lies within a certain interval about the estimated wavelet power. The estimated wavelet power is deemed statistically different from zero if the CI does not straddle zero. We replace the theoretical cross-wavelet power $\sigma_X\sigma_Y\sqrt{P_k^X P_k^Y}$ with the true cross-wavelet power $TW_n^2(s)$, then the confidence interval of $TW_n^2(s)$ is [1,3,5]

$$\frac{\nu|WCS_i(s)|}{\chi_\nu^2(p/2)} \leq TW_n^2(s) \leq \frac{\nu|WCS_i(s)|}{\chi_\nu^2(1-p/2)} \quad \text{(A4)}$$

where p is the given significance (p=0.05 for the 95% CI) and $\chi_\nu^2(p/2)$ representing the value of χ^2 at quantile $p/2$ and degree of freedom ν.

Using equation (A4), we can obtain the CIs for the peaks in a cross-wavelet power spectrum to compare against either the mean background or against other peaks.

Wavelet coherence is the square of the cross-spectrum normalized by the individual power spectra and measures the cross-correlation between two time series as a function of frequency. Therefore, we can obtain the CIs and then the significance test for wavelet coherence based on the equation (A4).

A.4. Graphical description

As shown in Figure 4, the black contour lines indicate the regions of power significant at the 5% level based on 1000 Monte Carlo simulations [1,5]. The curved line at the bottom is called the cone of influence (CoI) (the black curve) and below which, the wavelet can be subject to edge effects due to the use of a finite length series [1]. Therefore, to avoid spurious features caused by the wavelet method, only the information above the CoI is considered. A colour scale indicates if the periodicities have strong (red) or weak (blue) power for certain period and time, as shown in the right panel of Figure 4. The arrows show the phase difference between the weekly hospital notifications of A(H7N9) and meteorological factors: → in phase; ← in anti-phase; ↓:X leading Y by 90°; ↑: Y leading X by 90° [1,3–5].

B. Figures

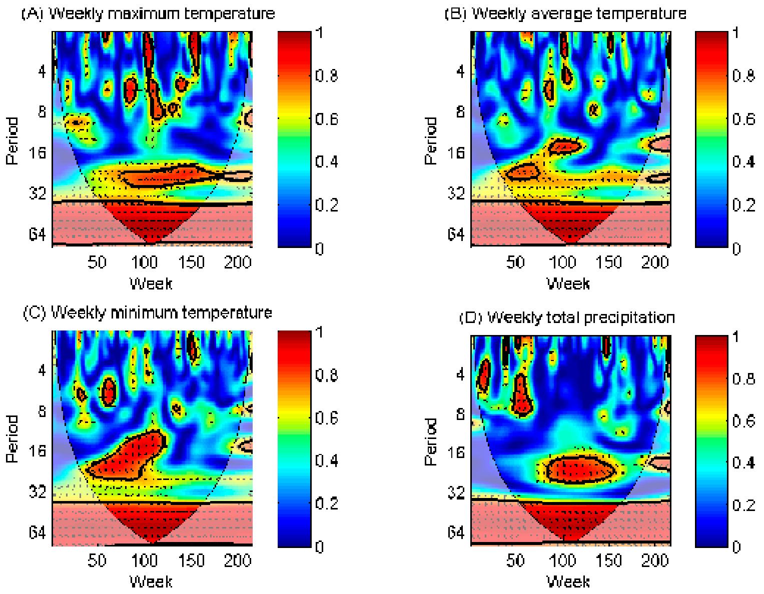

Figure S1. The wavelet coherence between weekly reported human cases of A(H7N9). (A) Weekly maximum temperature; (B) Weekly average temperature; (C) Weekly minimum temperature and (D) Weekly total precipitation for Guangdong province.

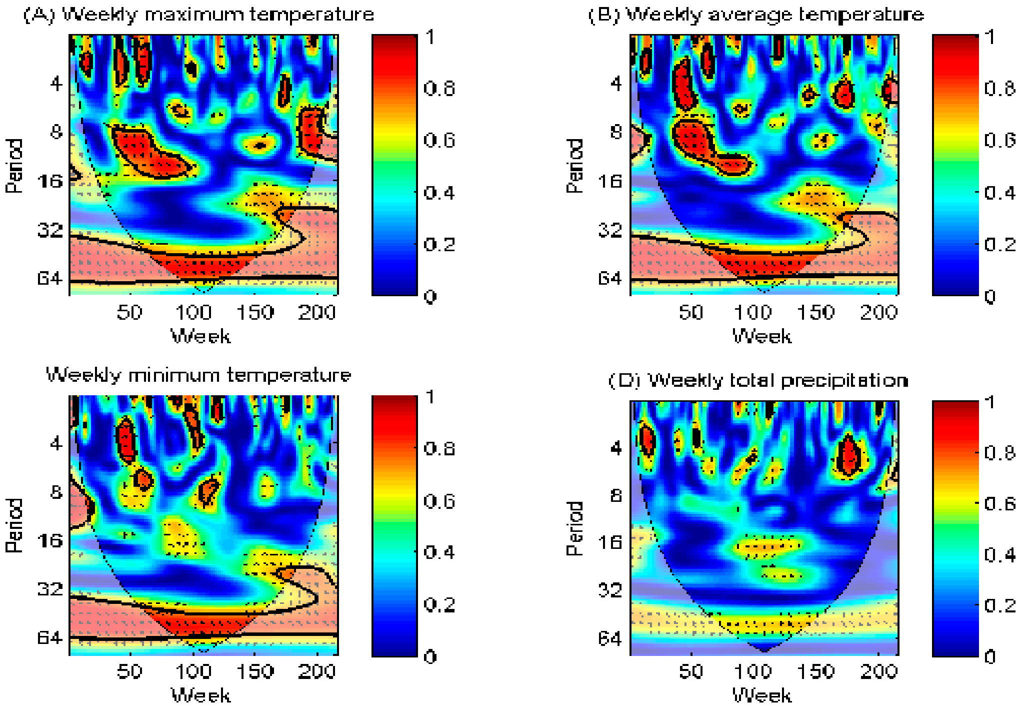

Figure S2. The wavelet coherence between weekly reported human cases of A(H7N9). (A) Weekly maximum temperature; (B) Weekly average temperature; (C) Weekly minimum temperature and (D) Weekly total precipitation for Jiangsu province.

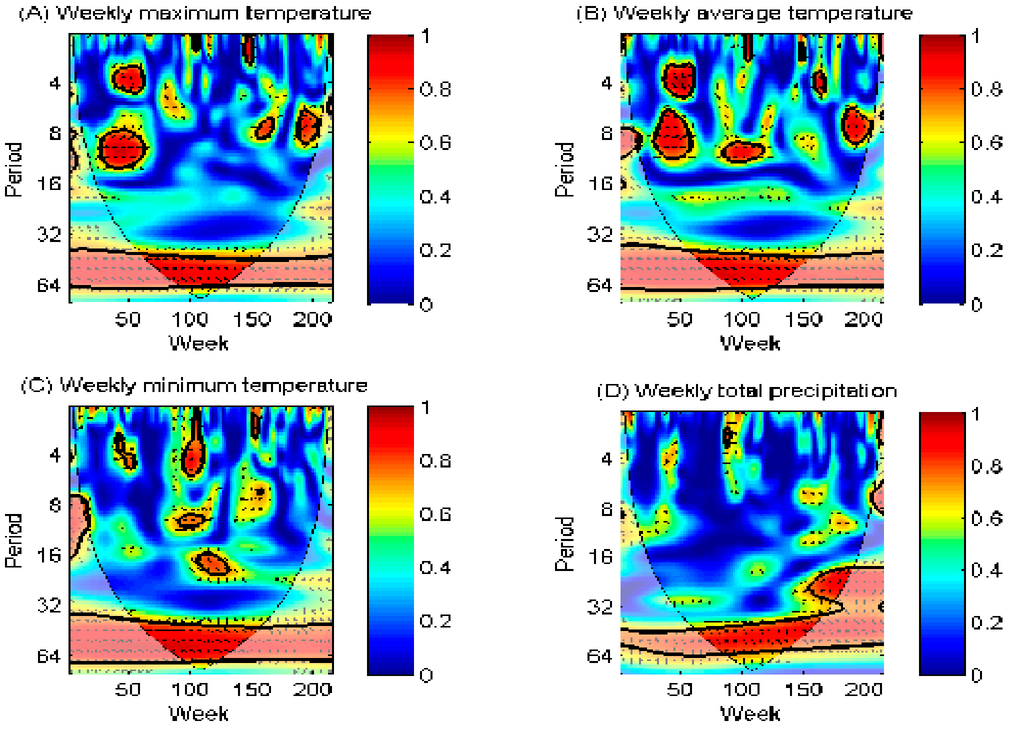

Figure S3. The wavelet coherence between weekly reported human cases of A(H7N9). (A) Weekly maximum temperature; (B) Weekly average temperature; (C) Weekly minimum temperature and (D) Weekly total precipitation for Fujian province.

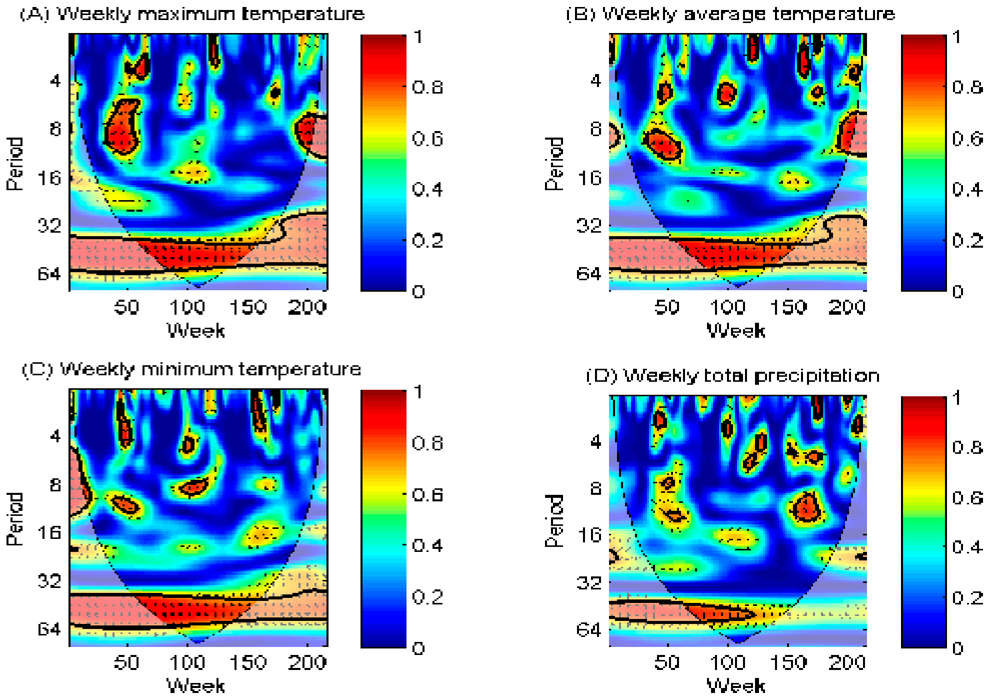

Figure S4. The wavelet coherence between weekly reported human cases of A(H7N9). (A) Weekly maximum temperature; (B) Weekly average temperature; (C) Weekly minimum temperature and (D) Weekly total precipitation for Anhui province.

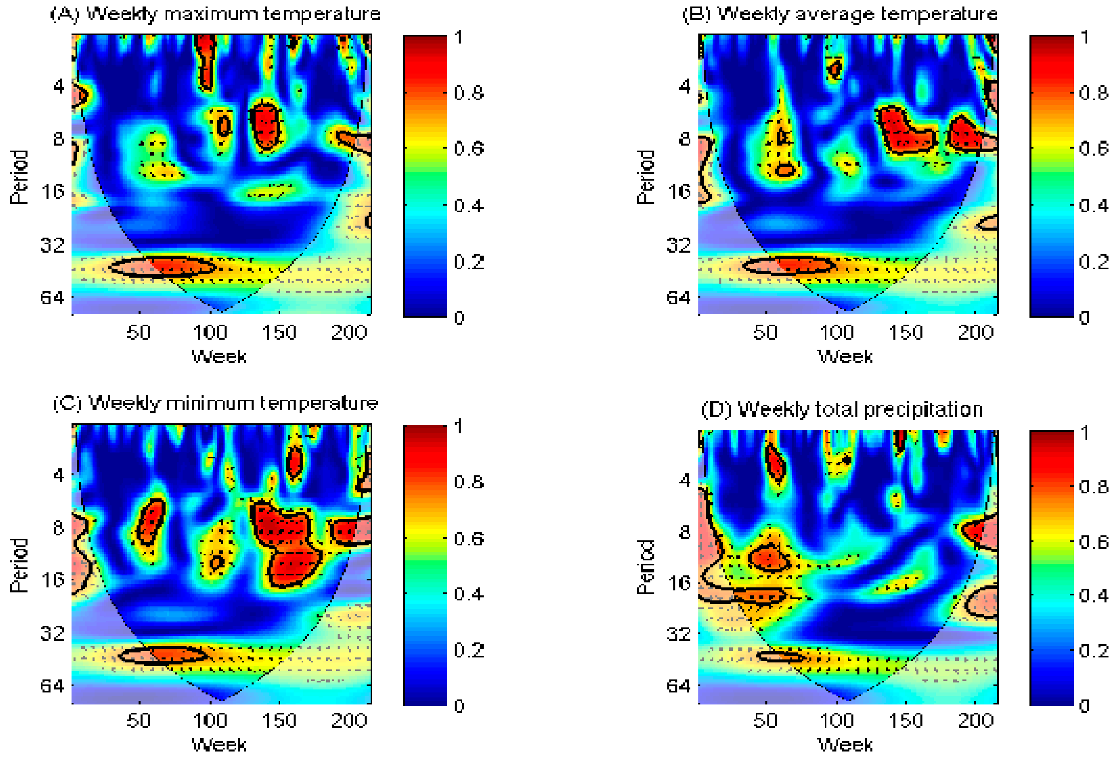

Figure S5. The wavelet coherence between weekly reported human cases of A(H7N9). (A) Weekly maximum temperature; (B) Weekly average temperature; (C) Weekly minimum temperature and (D) Weekly total precipitation for Jiangxi province.

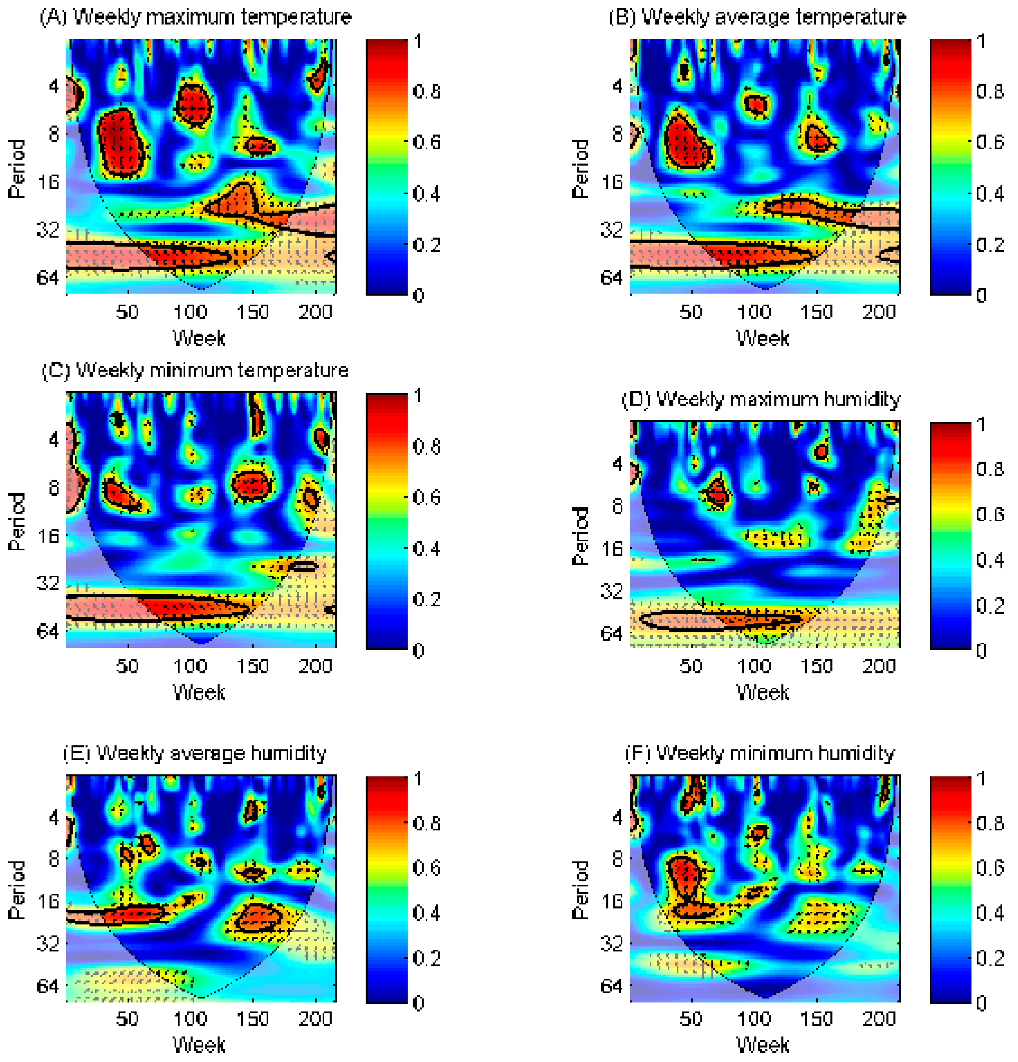

Figure S6. The wavelet coherence between weekly reported human cases of A(H7N9). (A) Weekly maximum temperature; (B) Weekly average temperature; (C) Weekly minimum temperature, (D) Weekly maximum relative humidity; (E) Weekly average relative humidity and (F) Weekly minimum relative humidity for Hunan province.

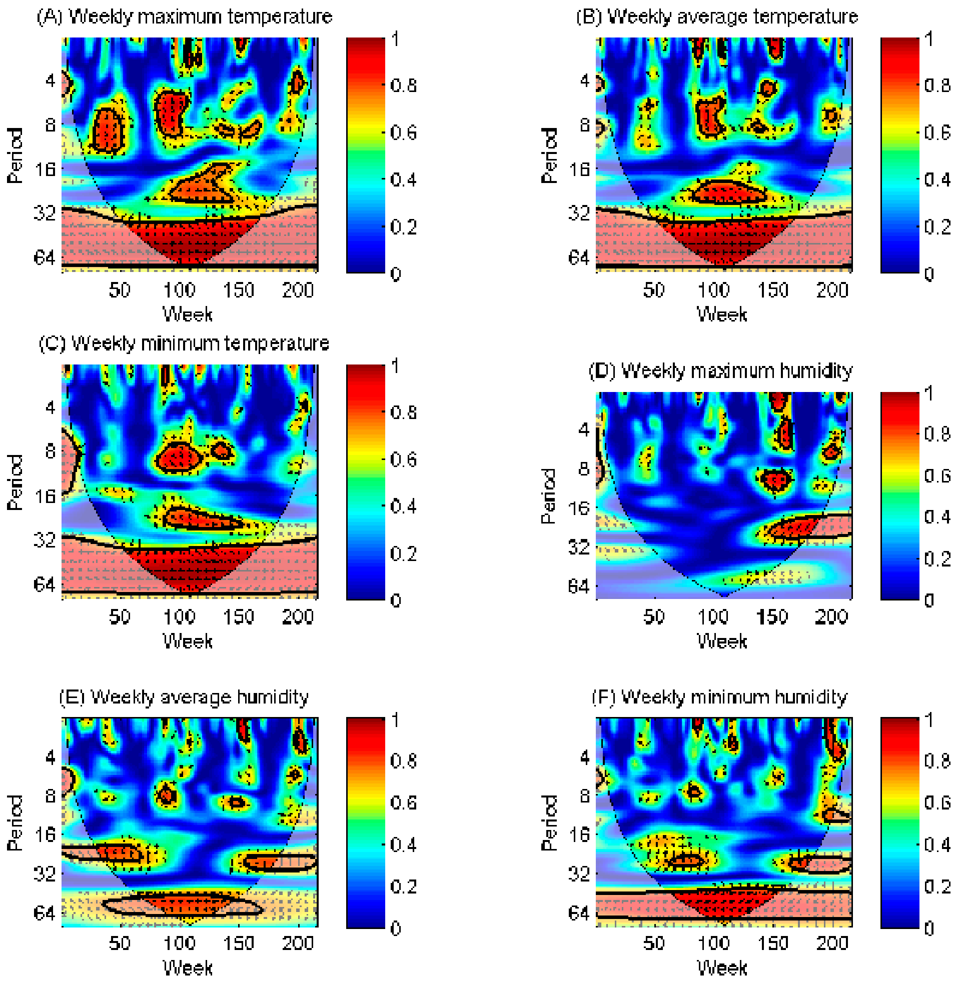

Figure S7. The wavelet coherence between weekly reported human cases of A(H7N9). (A) Weekly maximum temperature; (B) Weekly average temperature; (C) Weekly minimum temperature; (D) weekly maximum relative humidity; (E) Weekly average relative humidity and (F) Weekly minimum relative humidity for Shanghai province.

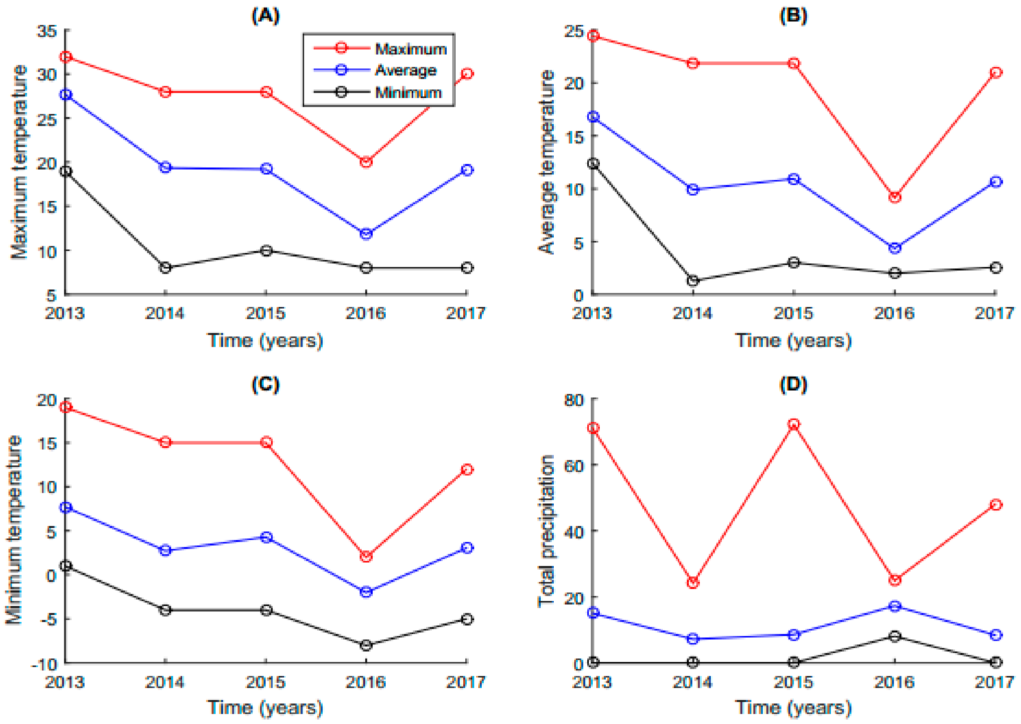

Figure S8. The plot of maximum, average, minimum temperature and total precipitation from the year 2013 to 2017. The temperature and total precipitation are taken out from Figure 3(B-E) in the time period of the five peaks corresponding to the maximum number of A(H7N9) cases in Figure 3(A). (A) Maximum temperature; (B) Average temperature; (C) Minimum temperature; (D) Total precipitation.

References

1. Torrence, C.; Compo, G.P. A practical guide to wavelet analysis. B. Am. Meteorol. Soc. **1998**, 79, 61-78.
2. Li, L.; Qian ,J.; Ou , C.Q. et al., Patial and temporal analysis of Air Pollution Index and its timescale-dependent relationship with meteorological factors in Guangzhou, China, 2001-2011, Environ Pollut **2014**, 190, 75-81.
3. Maraun, D.; Kurths, J. Cross wavelet analysis: significance testing and pitfalls}, Nonlin. Processes Geophys **2004**, 11, 505-514.
4. Diaz-Sandoval, R.; Erdelyi, R.; Maheswaran, R. Could periodic patterns in human mortality be sensitive to solar activity? Ann Geophys **2011**, 29, 1113-1120.
5. Grinsted, A.; Moore, J.C.; Jevrejeva, S. Application of the cross wavelet transform and wavelet coherence to geophysical time series, Nonlin. Processes Geophys **2004** , 11, 561-566.
6. Imbens, G. One-step estimators for over-identified generalized method of moments models, Rev of Econ Stud **1997**, 64, pp. 359-367.
7. Imbens, G. Generalized method of moments and empirical likelihood, J Bus Econ Stat 2002, 20, 493-506.
8. Lalonde, T.L.; Jeffrey, R.W.; Yin, J.Q. GMM logistic regression models for longitudinal data with time-dependent covariates and extended classifications, wileyonlinelibrary.com, DOI: 10.1002/sim.6273. /Eco-nometrics2_05_II /Slides/13_gmm_2pp.pdf.
9. Blundell, R.; Bond, S. Initial Conditions and Moment Restrictions in Dynamic Panel Data Models, J Econometrics **1998**, 87, 115-143.
10. Alastair, H. Generalized Method of Moments, OXFORD UNIV PR, **2005**, 1-61.

© 2019 by the authors. Submitted for possible open access publication under the terms and conditions of the Creative Commons Attribution (CC BY) license (http://creativecommons.org/licenses/by/4.0/).
